# Ca^2+^ Sensors Assemble: Function of the MCU Complex in the Pancreatic Beta Cell

**DOI:** 10.3390/cells11131993

**Published:** 2022-06-22

**Authors:** Jack G. Allen, Jeffery S. Tessem

**Affiliations:** Department of Nutrition, Dietetics, and Food Science, Brigham Young University, Provo, UT 84602, USA; jackga@byu.edu

**Keywords:** pancreatic β-cell, MCU complex, MICU1, MICU2, MICU3, EMRE, Ca^2+^ flux

## Abstract

The Mitochondrial Calcium Uniporter Complex (MCU Complex) is essential for β-cell function due to its role in sustaining insulin secretion. The MCU complex regulates mitochondrial Ca^2+^ influx, which is necessary for increased ATP production following cellular glucose uptake, keeps the cell membrane K^+^ channels closed following initial insulin release, and ultimately results in sustained insulin granule exocytosis. Dysfunction in Ca^2+^ regulation results in an inability to sustain insulin secretion. This review defines the functions, structure, and mutations associated with the MCU complex members mitochondrial calcium uniporter protein (MCU), essential MCU regulator (EMRE), mitochondrial calcium uptake 1 (MICU1), mitochondrial calcium uptake 2 (MICU2), and mitochondrial calcium uptake 3 (MICU3) in the pancreatic β-cell. This review provides a framework for further evaluation of the MCU complex in β-cell function and insulin secretion.

## 1. Ca^2+^ Dependent β-Cell Glucose Stimulated Insulin Secretion

Pancreatic endocrine hormones regulate systemic metabolism and are essential to preserving blood glucose homeostasis. These hormones are produced by groups of endocrine cells found in pancreatic islets of Langerhans. The islets are composed of alpha, beta, gamma, delta, and epsilon cells which secrete their specific hormone in response to various signals. The pancreatic endocrine hormones include pancreatic polypeptide, somatostatin, ghrelin, glucagon, and insulin [1]. The pancreatic β-cell secretes insulin in response to elevated blood glucose levels observed during the fed state. Insulin’s primary role is to induce the uptake of glucose by the muscle, adipose, and liver, thus decreasing circulating glucose levels and restoring euglycemia [2]. 

Mature insulin is formed in the β-cell and prepared for exocytosis. Each insulin granule contains roughly 250,000 molecules of insulin and has a diameter of 250 nm [3]. The mature insulin granules are exocytosed following stimulation via glucose (glucose stimulated insulin secretion, GSIS) [4]. Pancreatic GSIS begins with glucose uptake via a GLUT transporter [5]. The glucose is metabolized through glycolysis, the TCA cycle, and the electron transport chain to increase cellular ATP pools. Pyruvate kinase and mitochondrial oxidative phosphorylation increase the cytoplasmic ATP concentration, which induces the closure of K_ATP_ channels at the cell membrane [6]. The K_ATP_ channel closure alters the cell membrane electrochemical gradient, which stimulates the L-type Ca^2+^ channels and allows Ca^2+^ influx to the cytosol from the extracellular environment. Ca^2+^ is also released to the cytosol from the smooth endoplasmic reticulum, further increasing cytosolic Ca^2+^ concentration [7]. 

**Figure 1:**
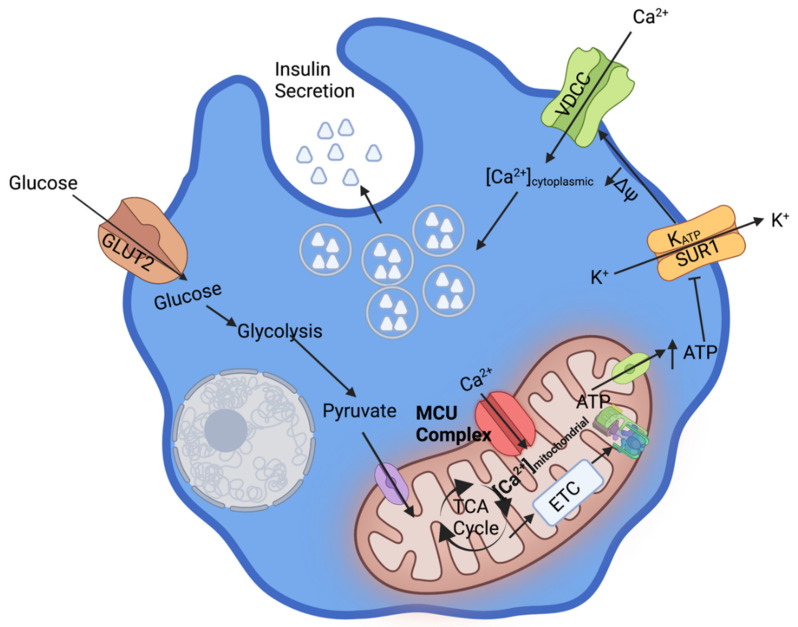
Mitochondrial Calcium Flux and GSIS in the Pancreatic β-cell. Under high blood glucose conditions, glucose enters the β-cell via the GLUT2 transporter. Glucose is then shunted to glycolysis, the TCA cycle, and the electron transport chain to generate ATP. ATP closes the K_ATP_ channels, which results in insulin granule exocytosis. Ca^2+^ is shunted to the mitochondria to further stimulate ATP production and cause continued insulin secretion. This figure was created with biorender.com.

Ca^2+^ entry through pancreatic β-cell membrane voltage-gated L-type Ca^2+^ channels triggers insulin granule exocytosis and is required for the postprandial spike in insulin secretion [8]. Ca^2+^ influx to the cytosol also stimulates metabolic enzymes which increase ATP production [9]. The initial insulin secretion is typically insufficient to restore euglycemia, causing continued glucose uptake via a GLUT transporter. Continued glucose uptake causes the β-cell to shunt Ca^2+^ to the mitochondria, through the MCU complex, to perpetuate insulin secretion [7]. This will continue until euglycemia is achieved and glucose is no longer taken into the β-cell (Figure 1).

During resting conditions, the β-cell mitochondrial Ca^2+^ concentration is similar to the cytosolic Ca^2+^ concentration. Following the closure of the plasma membrane voltage-gated L-type K^+^ channels cytosolic Ca^2+^ concentration rises significantly, stimulates insulin secretion, and begins to diffuse into the mitochondrial matrix through the Mitochondrial Calcium Uniporter (MCU) complex. The MCU complex tightly regulates the influx of mitochondrial Ca^2+^ to prevent apoptosis [10]. Increased Ca^2+^ concentration in the mitochondrial matrix further increases ATP synthase activity. ATP is transported to the cell membrane to ensure the closure of the L-type voltage-gated K^+^ channels and to provide for continued Ca^2+^ influx to the cytosol. The continued Ca^2+^ influx allows for sustained GSIS [11]. 

**Figure 2:**
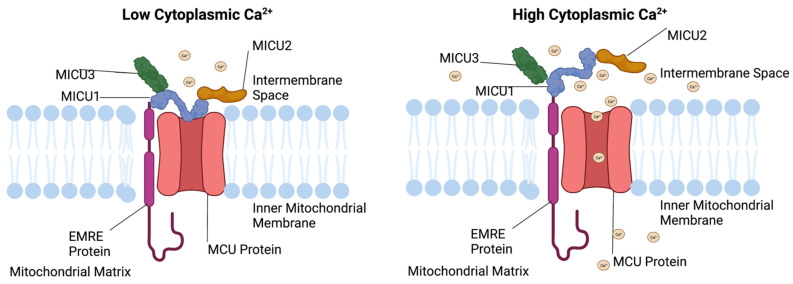
Mitochondrial Calcium Uniporter Complex under low and high cytosolic Ca^2+^ conditions. Under low cytosolic Ca^2+^ conditions the MCU is found in a closed state that impedes mitochondrial Ca^2+^ entry. Under elevated cytosolic Ca^2+^ conditions the MCU complex in the inner mitochondrial membrane is opened through Ca^2+^ binding, thus resulting in mitochondrial Ca^2+^ entry. MCU-Mitochondrial Calcium Uniporter, MICU1—Mitochondrial calcium uptake 1, MICU2—Mitochondrial calcium uptake 2, MICU3—Mitochondrial calcium uptake 3, and EMRE-essential MCU regulator. This figure was created with biorender.com.

The MCU complex is made up of five distinct proteins that detect cytoplasmic Ca^2+^ concentrations and regulate mitochondrial Ca^2+^ uptake. The tight regulation of mitochondrial and cytoplasmic Ca^2+^ concentrations maintain insulin secretion. These proteins are the mitochondrial calcium uniporter (MCU), mitochondrial calcium uptake 1 (MICU1), mitochondrial calcium uptake 2 (MICU2), mitochondrial calcium uptake 3 (MICU3), and the essential MCU regulator (EMRE) [12] (Figure 2). MICU1 and MICU2 detect fluctuations in cytosolic Ca^2+^ levels and MICU1 opens the MCU to allow mitochondrial Ca^2+^ influx, MICU2 has an inhibitory effect on the MCU complex under low cytoplasmic Ca^2+^ conditions (Figure 2). EMRE senses mitochondrial matrix Ca^2+^ levels and terminates Ca^2+^ influx. This review will focus on the MCU complex, and will present the function, location, and disorders associated with each of the MCU complex members and its role in pancreatic β-cell GSIS. 

## 2. Mechanisms of Mitochondrial Ca^2+^ Uptake 

Ca^2+^ influx into the mitochondria from the cytosol is driven by the highly negative electrochemical gradient (ΔΨ, ~−180 mV) [13,14], and the cytosolic Ca^2+^ concentration. The highly negative electrochemical potential is due to the electron transport chain pumping hydrogen ions into the intermembrane space [15]. Several experiments have investigated the relationship between cytosolic Ca^2+^ concentrations and mitochondrial matrix Ca^2+^ concentrations. Rizzuto et al. examined the effect of agonist driven changes in Ca^2+^ concentration. They showed that increased cytosolic Ca^2+^ concentration will inevitably lead to increased mitochondrial matrix Ca^2+^ concentration. However, in the presence of uncoupling proteins in the inner mitochondrial membrane, there is no net diffusion of Ca^2+^ into the mitochondrial matrix [16].

Cellular Ca^2+^ influx has the potential to induce β-cell GSIS or apoptosis. The subsequent mitochondrial signals differ due to the polyphosphate molecules located in the mitochondrial matrix [17]. The mitochondria can function as a Ca^2+^ sink due to these polyphosphate molecules [16]. Experiments that decrease mitochondrial matrix polyphosphate levels impair the mitochondria’s ability to sustain high matrix Ca^2+^ levels without signaling for apoptosis [16]. The MCU and polyphosphate are both essential to Ca^2+^ regulation, and studies are needed to examine the relationship between these two methods of altering mitochondrial Ca^2+^ flux. 

Mitochondrial matrix Ca^2+^ influx regulates mitochondrial fuel metabolism. Increased matrix Ca^2+^ levels directly stimulate pyruvate dehydrogenase phosphatase [18], isocitrate dehydrogenase, and α-ketoglutarate dehydrogenase [19]. Other studies have shown that increased mitochondrial matrix Ca^2+^ levels increase electron transport chain (ETC) activity. Rutter et al. showed that increased matrix Ca^2+^ specifically stimulated ETC complexes I, III, and IV. Stimulation resulted in greater conductance and fuel transport [20]. Increased metabolism associated with increased matrix Ca^2+^ concentration results in increased ATP production and sustained cell membrane voltage-gated potassium channel closure [21]. These effects lead to enhanced GSIS [11]. Excess matrix Ca^2+^ concentration results in the formation of calcium phosphate molecules. Calcium phosphate decreases the activity of ATP synthase activity by blocking the interaction between ETC complex I and NADH [22]. 

The sodium calcium exchanger (NCLX), and sarcoplasmic reticulum/endoplasmic reticulum Ca^2+^ ATPase (SERCA) pumps provide the majority of mitochondrial Ca^2+^ efflux [23]. NCLX and MCU work in conjunction to balance Ca^2+^ needs in the mitochondria. NCLX activity is regulated by concentration gradients across the inner mitochondrial membrane [24]. Sodium concentration is high in the intermembrane space, and Ca^2+^ concentration is higher in the mitochondrial matrix, in pancreatic β-cells, following glucose stimulation. The increased matrix Ca^2+^ concentration causes increased ATP production and is needed for insulin secretion. NCLX activity following insulin secretion utilizes the sodium and Ca^2+^ concentration gradients to pump Ca^2+^ out of the cell and restore basal inner mitochondrial membrane resting potential [23,25]. Pumping Ca^2+^ out of the matrix will prepare the cell for future GSIS. 

Cellular signals for apoptosis utilize increased mitochondrial matrix Ca^2+^. Mitochondrial Ca^2+^ influx can lead to either apoptosis or GSIS. Mitochondrial membrane permeability transition pore (PTP) opening allows for rapid Ca^2+^ release from the mitochondria into the cytosol. When the PTP is constitutively open mitochondrial metabolism decreases, the mitochondrial membrane ruptures, and apoptogenic proteins are released from the mitochondria [26,27]. During apoptosis, apoptogenic proteins facilitate rough endoplasmic reticulum (RER) Ca^2+^ release into the cytosol. RER Ca^2+^ release into the cytoplasm in conjunction with PTP opening results in cellular apoptosis [24,25]. 

The MCU complex is the primary method of Ca^2+^ influx into the mitochondrial matrix. In the β-cell, this is essential for maintaining GSIS [28]. The MCU complex is the channel by which cytosolic Ca^2+^ diffuses to enter the mitochondria (Figure 2). Increased β-cell glucose concentrations stimulates mitochondria mediated ATP production, and increases transcription and translation of the MCU protein [29,30]. Therefore, chronic glucose exposure increases MCU protein levels in the inner mitochondrial matrix. Increased MCU levels under glucotoxic conditions results in increased levels of reactive oxygen species (ROS) and increased apoptosis levels. These effects were negated when the MCU was inhibited using ruthenium red, a common MCU complex inhibitor [29]. Additional studies indicate that a loss of function in mitochondrial Ca^2+^ flux proteins, MCU and NCLX have been observed in some type 2 diabetes patients. This phenomenon requires further research [31]. This review will examine the structure, function, and diseases associated with each of the components of the MCU complex. 

## 3. MCU Complex in the β-Cell

The MCU complex is essential for proper mitochondrial calcium uptake. This function is due to sensing the cytosolic and mitochondrial matrix Ca^2+^ concentration. The MCU complex is composed of five distinct portions MCU, MICU1, MICU2, MICU3, and EMRE. Under conditions of high cytosolic Ca^2+^ concentrations MICU1 is stimulated and the MCU opens, allowing mitochondrial Ca^2+^ influx. As the cytosolic Ca^2+^ concentration decreases, the MCU complex is closed due to MICU2 activation (Figure 2). Finally, when mitochondrial Ca^2+^ concentrations are elevated, a conformational change in the EMRE occurs that blocks continued mitochondrial Ca^2+^ entry. 

Initial β-cell MCU knockout experiments have provided knowledge on MCU complex function in human tissues. In 2020 a β-cell specific MCU complex knockout mouse provided insight into β-cell specific MCU function. These mice produced insulin but had impaired first phase insulin secretion and could not sustain insulin secretion [32]. These results and cell line research indicate that the MCU complex is involved in the first and second phase insulin secretion in the β-cell [32,33]. 

MCU complex function was also tested in human insulin-secreting cell lines. These cell lines were exposed to a known MCU simulant (kaempferol) or to a known inhibitor (mitoxantrone). Kaempferol exposure resulted in increased mitochondrial Ca^2+^ flux and a 70% increase in insulin secretion. The increased Ca^2+^ flux did not have cytotoxic effects. Mitoxantrone exposure decreased mitochondrial Ca^2+^ flux and resulted in decreased total insulin secretion and insulin release was not potentiated following glucose exposure [34]. These data indicate the MCU complex has an essential role in sustained insulin secretion [32,33,34]. The known functions of MCU Complex Components in the β-cell are defined in Table 1. In the following sections, we will describe the function and structure of each component of the MCU complex. 

### 3.1. MCU Protein Function

The MCU protein is a mitochondrial Ca^2+^ channel that is necessary for first and second phase β-cell insulin secretion. MCU protein knockout blocks Ca^2+^ transfer to the mitochondrial matrix. Knockout of the MCU protein results in decreased Ca^2+^ dependent activity of the electron transport chain and ATP synthase, and ultimately reduces cellular ATP levels [21,40]. β-cell MCU protein deletion impairs insulin secretion due to ATP concentrations dropping below the necessary threshold to close the plasma membrane K_ATP_ channels [35,41]. These results show that the MCU protein is essential for β-cell ATP synthesis and GSIS [42]. The MCU protein is currently being examined as a target for pharmacological activation in patients with type 2 diabetes (T2D). Researchers have recorded dysregulated mitochondrial Ca^2+^ flux in T2D patients, and MCU activation may help alleviate T2D symptoms [36]. 

The MCU protein is specific for Ca^2+^ entry due to channel size and protein structure. The MCU protein is Ca^2+^ inwardly rectifying, due to Ca^2+^ binding site specificity. These binding sites have an extremely high affinity for Ca^2+^ (dissociation constant less than 2 nM) which causes the MCU protein to exhibit Ca^2+^ sensitivity even at extremely low cytoplasmic Ca^2+^ concentrations [43]. The MCU protein Ca^2+^ binding site specificity prevents MCU protein activation by any other ion. The MCU operates independently and is not coupled to the transport of any other ion [44]. In the pancreatic β-cell the increase in cytoplasmic Ca^2+^ concentration following cellular glucose uptake and metabolism results in a Ca^2+^ concentration gradient between the cytoplasm and the mitochondrial matrix. This gradient drives the selective Ca^2+^ diffusion into the mitochondrial matrix by way of the MCU protein [45].

Palmitate induces endoplasmic reticulum (ER) Ca^2+^ efflux, leading to ROS formation in β-cells. Palmitate induces a positive feedback loop with ER Ca^2+^ depletion and ROS generation that leads to cell death [46,47]. Exposure to palmitate, as is observed in T2D with elevated free fatty acids, upregulates the expression of MCU protein as a protective mechanism. Increased MCU production provides a Ca^2+^ sink and prevents mitochondrial ROS generation, which is induced by greater fatty acid metabolism [48,49]. Palmitate also increases MCU protein activity, though the mechanism is unknown. The MCU protein protects the β-cell from cell death during excessive palmitate exposure. 

β-cell Na^+^ channels have a regulatory role on MCU function. Mouse islets were used to clarify the relationship between the two regulatory channels. It was already known that MCU protein knockout is associated with increased mitochondrial fragmentation, decreased oxidative phosphorylation, impaired glycolysis, and impaired proliferation [50]. Researchers performed a selective β-cell Na^+^ channel knockout, and the cells were unable to sustain insulin secretion. Cytosolic Na^+^ influx activates the MCU and allows for increased mitochondrial Ca^2+^ influx. Na^+^ then exits the mitochondria via NCLX [51], and is exchanged for Ca^2+^, further increasing mitochondrial Ca^2+^ concentration and aiding in GSIS. 

### 3.2. MCU Protein Structure

The MCU is a 40 kilodalton protein located in the inner mitochondrial membrane [52]. The MCU protein contains two transmembrane helices, which are separated by a linker that reaches the intermembrane space. This linker contains acidic amino acids, which are required for MCU activity [53]. The MCU has a tetrameric form with soluble and transmembrane domains forming symmetric arrangements in the channel. Ca^2+^ specificity is determined through two acidic rings along the central axis of the Ca^2+^ pore [54]. Chanel size and acidic amino acids in the protein core also grant Ca^2+^ specificity [55].

The MCU transmembrane helices are essential for Ca^2+^ specificity and MCU function. When a mutant MCU expresses only one helix, Ca^2+^ uptake is terminated. Single nucleotide polymorphisms (SNPs) resulting in bulky side chains disrupt the helix structure and also cause a lack of function. These results show the essential role of the transmembrane helices in Ca^2+^ transport across the inner mitochondrial membrane [56]. The loss of function was consistent in models with and without the regulatory subunits EMRE and MCUR1 suggesting that the issue is in the MCU protein, not in MCU complex regulatory subunits [56].

The *MCU* gene is on human chromosome 10 [57]. The MCU protein is highly conserved across organisms, which highlights the essential physiological role of this protein. No MCU mutations that significantly alter protein function have been linked to human diseases, suggesting that major mutations may confer embryonic lethality [58]. 

### 3.3. MICU1 Function

**Figure 3:**
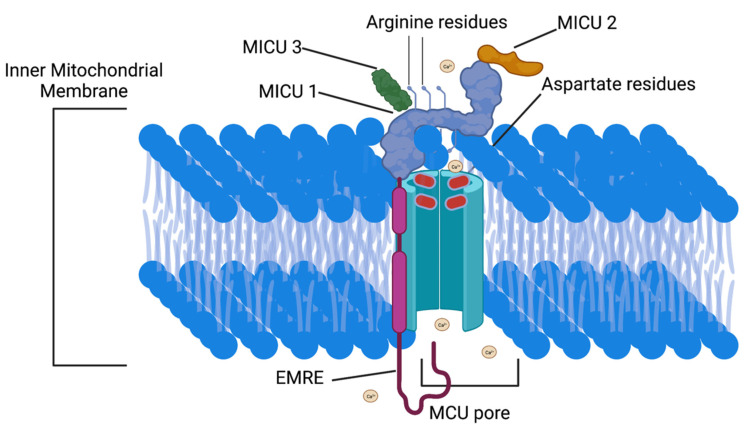
Mitochondrial Calcium Uniporter and MICU1 interaction under high cytosolic Ca^2+^ conditions. Under high cytosolic Ca^2+^ conditions the MCU complex opens to allow mitochondrial Ca^2+^ influx. The MCU complex opens due to interactions between MCU protein aspartate residues and the cytosolic Ca^2+^. When cytosolic Ca^2+^ is low the MICU1 arginine fingers bind to the MCU protein aspartate residues and closes the MCU complex. MCU-Mitochondrial Calcium Uniporter, MICU1—Mitochondrial calcium uptake 1. This figure was created with biorender.com.

Mitochondrial calcium uptake 1 (MICU1) functions as a regulatory subunit in the MCU complex. MICU1 is required to preserve normal mitochondrial Ca^2+^ levels [59]. During periods of normal physiologic Ca^2+^ concentrations, MICU1 closes the MCU by binding to the MCU acidic aspartate residues in the intermembrane space to close the MCU pore. MICU1 contains arginine finger motifs that bind with the MCU aspartate rings to ensure MCU complex closure (Figure 3). Closing the MCU channel decreases Ca^2+^ flux into the mitochondrial matrix [60]. This phenomenon was observed in human β-cells, hepatocytes, HeLa cells, and in mouse hepatocytes [35,59,60,61].

When cytosolic Ca^2+^ levels rise, such as following β-cell glucose uptake, Ca^2+^ diffuses through the outer mitochondrial membrane and interacts with MICU1 and the MCU. Ca^2+^ binds the MCU aspartate rings, resulting in the dissociation of MICU1 from MCU [60]. MICU1 dissociation allows Ca^2+^ to flow into the mitochondrial matrix, which stimulates ATP production and ultimately leads to sustained GSIS in the β-cell [35]. MICU1 knockout in β-cells results in impaired Ca^2+^ uptake. This impaired Ca^2+^ uptake resulted in structural changes including forming short tubular mitochondria. MICU1 knockout was not rescued by increased expression of MCU [35,37,62,63].

MICU1 also has a regulatory role in preventing excessive mitochondrial matrix Ca^2+^ influx. MICU1 deletion in HeLa cell lines leads to rapid apoptosis due to excessive mitochondrial matrix Ca^2+^ influx. MICU1 knockout removes the primary gatekeeper protein on the MCU and unregulated mitochondrial Ca^2+^ influx causes apoptosis [37]. The majority of MICU1 studies utilized a mouse model or HeLa cells. Further experimentation is needed to fully understand the role of MICU1 on apoptosis in the β-cell. 

### 3.4. MICU1 Structure

MICU1 is a 54 kilodalton protein that is made up of 476 amino acids. MICU1 is connected to the MCU complex via EMRE. EMRE spans the inner mitochondrial membrane and extends into the intermembrane space to anchor MICU1 to the protein complex [37]. MICU1 forms a heterodimer with MICU2 in low cytosolic Ca^2+^ conditions. This heterodimer then binds to the surface of the MCU and prevents Ca^2+^ diffusion into the mitochondrial matrix [64]. 

The *MICU1* gene is located on human chromosome 10 in close proximity to the MCU gene [57]. Expression of MICU1 and MICU2 have a positive linear relationship. MICU1 overexpression results in increased MICU2 expression, but overexpression of MICU2 does not result in MICU1 overexpression [64,65]. 

### 3.5. MICU2 Function

MICU2 forms a disulfide bond with MICU1 and is involved in Ca^2+^ sensing in conjunction with MICU1. β-cell siRNA mediated MICU2 knockdown attenuates GSIS by 41–51 percent [38]. Similarly, mitochondrial Ca^2+^ uptake is attenuated, the ETC is not stimulated, and ATP is not transported to the cell membrane to close the K^+^ channels. These data indicate that MICU2 is essential for sustained GSIS in the pancreatic β-cell. MICU2 deletion results in Ca^2+^ accumulation in the submembrane compartment in the mitochondrial matrix of the β-cell [38].

MICU2 also plays a role in cytosolic Ca^2+^ regulation in the β-cell. In β-cell MICU2 knockout mice GSIS was inhibited by 57%, and there was decreased activity at the cell membrane Ca^2+^ channels. There is currently no known mechanism for the decreased cytosolic Ca^2+^ levels associated with MICU2 knockout. There is also an unknown mechanism by which MICU2 knockout mice were able to sustain euglycemia with decreased insulin secretion. There was also an accumulation of insulin within the mouse β-cells. Defining the mechanism by which MICU2 knockout impairs cytosolic β-cell Ca^2+^ levels is imperative.

MICU1 and MICU2 have complementary roles. When cytosolic Ca^2+^ levels are elevated, Ca^2+^ binds to MICU2 and removes the inhibitory effect on the MCU complex [66]. This causes MICU1 dissociation from the MCU protein and Ca^2+^ influx. Conversely, when cytosolic Ca^2+^ levels are decreased, MICU2 binds to MICU1 and causes blockage of the MCU protein-mediated Ca^2+^ influx. When MICU1 is silenced in rodent models MICU2 activity overexpression does not rescue the MICU1-mediated mitochondrial deficiency [64]. Similarly, when MICU2 is knocked out and MICU1 is functional, mitochondrial Ca^2+^ uptake is attenuated [38]. Knockout of either MICU1 or MICU2 results in decreased MCU complex size and function. These data clarify that both MICU1 and MICU2 are essential for MCU function, and MICU2 primarily functions as an intermembrane space Ca^2+^ sensor [64,67]. Both MICU1 and MICU2 knockout result in decreased insulin secretion and an inability to sustain GSIS [38].

MICU1 and MICU2 function in conjunction to regulate Ca^2+^ influx to the mitochondrial matrix in the β-cell [67]. Most of the research on MICU2 does not take place in β-cells. Therefore, further research is needed to fully understand the regulatory role of MICU2 on mitochondrial Ca^2+^ flux in β-cells. 

### 3.6. MICU2 Location

MICU2 expression varies based on the tissue type. MICU2 localizes exclusively to mitochondria. Though MICU2 is found throughout the body, expression is highest in the visceral organs [68]. The *MICU2* gene is located on chromosome 13. Though this gene is on a different chromosome than MICU1 there is a positive linear relationship between the expression of MICU1 and MICU2 expression [57]. This suggests coordinated transcription of these proteins. 

### 3.7. MICU3 Function

MICU3 is an EF hand containing protein that forms a disulfide bond with MICU1 and has no association with MICU2. MICU3 functions as an enhancer of mitochondrial Ca^2+^ uptake via the MCU [69] (Figure 2). MICU3 enhances MCU opening in response to rapid increases in cytosolic Ca^2+^ concentration [70]. In this regard MICU3 functions as a regulatory protein in the MCU complex in human tissues. 

MICU3 assists in cell-mediated apoptosis. MICU3 silencing in vivo conferred cardioprotective effects in mice with induced cardiac dysfunction. Induced cardiac dysfunction normally results in apoptosis via Ca^2+^ overload in the mitochondria [71]. MICU3 knock-out mice blocked extreme mitochondrial Ca^2+^ influx and prevented cardiac apoptosis in rodents. Though these data pertain to heart function, this same mechanism induces apoptosis in pancreatic β-cells [70]. Further research must be performed to clarify the anti-apoptotic effects of MICU3 knockout on pancreatic β-cells. 

### 3.8. MICU3 Location

MICU3 is localized to the outer leaflet of the mitochondrial inner membrane and is expressed throughout the body. The highest concentrations of MICU3 are found in the brain and skeletal muscle [72]. The *MICU3* gene is located on chromosome 8 [57].

### 3.9. EMRE Function

**Figure 4:**
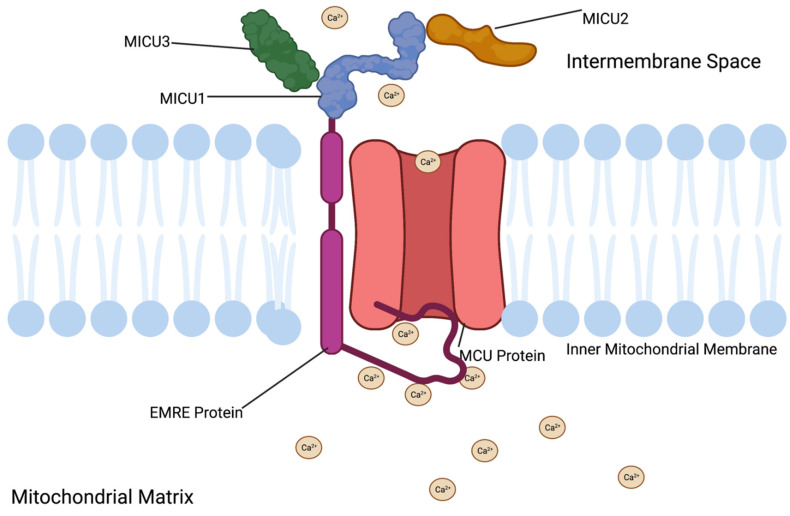
Mitochondrial Calcium Uniporter Complex under high mitochondrial matrix Ca^2+^ conditions. Increased mitochondrial matrix Ca^2+^ interacts with the EMRE tail that extends into the mitochondrial matrix. This interaction causes a conformational change that results in the EMRE tail blocking the MCU complex. This interaction prevents further influx of Ca^2+^ into the mitochondria. MCU-Mitochondrial Calcium Uniporter, MICU1—Mitochondrial calcium uptake 1, MICU2—Mitochondrial calcium uptake 2, MICU3—Mitochondrial calcium uptake 3, and EMRE-essential MCU regulator This figure was created with biorender.com.

The essential MCU regulator protein, EMRE, is a 10-kilodalton transmembrane protein. This protein is located in the inner mitochondrial matrix and is essential for matrix Ca^2+^ sensing [73,74]. EMRE’s N-terminal tail extends into the mitochondrial matrix and contains acidic amino acid residues that interact with matrix Ca^2+^ and change the EMRE protein conformation according to Ca^2+^ concentration variations (Figure 4). When matrix Ca^2+^ exceeds levels needed for cellular function Ca^2+^ binds to the acidic residues, an EMRE conformational change is induced and the MCU complex is closed. Low matrix Ca^2+^ concentration following periods of MCU closure causes Ca^2+^ to dissociate from the acidic residues of the EMRE N-terminal tail. Ca^2+^ dissociation results in a conformation change to EMRE that opens the MCU complex from the matrix side [74,75]. Therefore, mitochondria are protected from low matrix Ca^2+^ conditions and high matrix Ca^2+^ conditions via EMRE mediated Ca^2+^ sensing. This research was performed in human embryonic kidney cell lines, though this same conformational change is anticipated to occur in β-cells. 

EMRE post translational modifications, such as phosphorylation of serine residues S57 and S92, determine MCU localization in the mitochondrial membrane [39]. β-cell mitochondria are closely localized to the endoplasmic reticulum. This localization facilitates the uptake of mitochondrial Ca^2+^ via MCU following cellular glucose uptake [76]. Glucose uptake stimulates Ca^2+^ release from the endoplasmic reticulum. MICU1 exerts an excitatory effect on the MCU leading to Ca^2+^ uptake and GSIS [8]. EMRE post translational modifications cause the localization of the β-cell MCU-EMRE-MICU1-MICU2 complex to rest on the inner boundary region instead of along the invaginated cristae [39]. This positioning facilitates rapid GSIS following an excitatory stimulus in β-cells. The majority of the research on EMRE utilizes human embryonic kidney cells or HeLa cells. Further research is needed in β-cells to determine the effects of β-cell EMRE knockout. 

### 3.10. EMRE Location

EMRE spans the inner mitochondrial membrane in many cell types throughout the human body. Expression does not vary significantly according to tissue type. The *SMDT1* gene, which codes for EMRE, is located on chromosome 22 [57]. 

## 4. MCU Complex and Disease

The MCU complex is essential for sustained insulin secretion, though there are mutations in this complex that result in disease states. There are identified disease states for MCU protein, MICU1, and EMRE. There are currently no disease states identified for MICU2 and MICU3, suggesting the need for further research into these proteins. There is also a possibility that mutations in MICU2 and MICU3 result in spontaneous abortion. 

### 4.1. MCU Mutations

Mice lacking the MCU protein are viable, though there is an increase in embryonic spontaneous abortion. MCU knockout was also hypothesized to decrease apoptosis, however, mice models are inconclusive [77]. These mice exhibit decreased pyruvate dehydrogenase activity and impaired mitochondrial Ca^2+^ uptake. Though mice lacking the MCU protein are viable they are unable to increase mitochondrial metabolism in response to cellular signals [77,78]. 

Mutations in the inner mitochondrial membrane proteases result in altered MCU protein function. The mitochondrial protease m-AAA will degrade the MCU regulatory subunit EMRE when mutated. Protease m-AAA stimulated EMRE degradation results in constitutively active MCU channels facilitating Ca^2+^ overload and cell death [79]. MCU knockout models also conferred cardioprotective effects in mice models. Mice treated with histidine triad nucleotide binding 2, a known MCU antagonist, had attenuated cardiac microvascular ischema-reprefusion injuries [79,80,81]. 

### 4.2. MICU1 Mutations

Homozygous deletion of a 2755 base pair segment of exon 1 of MICU1 results in total deletion of the MICU1 protein. This mutation was observed in 2 human patients, who were tested for MICU1 function following an appointment with a physician. These patients presented with fatigue, migraine, and increased creatine kinase levels. The patients had decreased pyruvate dehydrogenase activity and were able to secrete low levels of insulin [82].

MICU1 loss of function mutation also presented as myopathy with extrapyramidal signs. This condition is characterized by abnormal Ca^2+^ handling. The phenotype consists of proximal myopathy, learning disabilities, and an extrapyramidal learning disorder [83]. 

### 4.3. MICU2 Mutations

A MICU2 truncation mutation was recently discovered in patients presenting with a severe neurological disorder and cognitive impairment. Samples were taken from patients and mitochondrial Ca^2+^ flux was measured. These cells had increased mitochondrial Ca^2+^ flux as compared to control cells [84]. 

### 4.4. EMRE Mutations

There are no known EMRE mutations in humans. The highly conserved nature of this protein is indicative of essential function. Interestingly, EMRE knockout mice are viable. Though these mice are viable they cannot utilize Ca^2+^ to stimulate the mitochondria and there is a very high rate of spontaneous abortion. Knockout mice are born at one-fifth the rate predicted by Mendelian genetics [85]. A summary table of all known mutations of MCU complex components and the associated effects are listed in Table 2.

## 5. Conclusions

The MCU complex is essential for sustained GSIS in the pancreatic β-cell [3,7,86,87]. Glucose uptake results in an increase in cytosolic Ca^2+^ concentrations, which causes Ca^2+^ to enter the intermembrane space and bind to MICU1 [37]. MICU1 activation opens the MCU channel and allows Ca^2+^ to flow into the mitochondria down its concentration gradient [71]. EMRE spans the inner mitochondrial matrix and binds MICU1, ensuring that MICU1 successfully closes MCU in low Ca^2+^ conditions [59,75]. EMRE contains acidic residues in the mitochondrial matrix that interact with Ca^2+^ in the matrix [87]. When Ca^2+^ levels are sufficient to sustain insulin secretion EMRE causes MICU1 to close the MCU channel and prevent excessive Ca^2+^ accumulation [75,86]. Under physiological conditions and in the absence of glucose MICU2 has an inhibitory effect on the MCU complex. Low intermembrane space Ca^2+^ activates MICU2, which causes a MICU1-MICU2 heterodimer to close the MCU channel and stop Ca^2+^ flow into the mitochondrial matrix [65,66]. Each of the MCU complex subunits have a Ca^2+^ sensing role in addition to providing the channel for Ca^2+^ to diffuse down. The MCU complex facilitates insulin secretion by increasing the flux of Ca^2+^ into the mitochondrial matrix. 

This review provides a framework for further studies to investigate MCU function in β-cells in various disease states, such as diabetes mellitus. These studies demonstrate the importance of the MCU in maintaining the ability of the β-cell to dynamically respond to elevated blood glucose through the propagation of insulin secretion. Given the effects of MCU component deletion and mutations on β-cell insulin secretion, these data suggest that the MCU complex may be pharmacologically targetable to modulate insulin secretion in patients with T2D. While much is currently known about various components in the β-cell, additional studies are needed to fully explore the role of this complex and its components in maintaining and improving functional β-cell mass. While studies have been completed that help to define the role of MCU, MICU1, and MICU2 in the β-cell, targeted studies on MICU3 and EMRE in the β-cell are still needed. These targeted studies on MICU3 and EMRE should include β-cell specific knock-out studies. In addition, the effects of naturally occurring MICU3 and EMRE mutations should be explored. The potential anti-apoptotic effects of MICU3 need to be explored to define a mechanism of action. Further research is also needed to understand the regulatory role of MICU2 on mitochondrial Ca^2+^ flux in β-cells. Finally, it is of extreme importance to understand how the MCU complex functions under various physiological states. Therefore, studies that explore the function of the wild type or mutant MCU complex components during diet-induced obesity, aging, pregnancy, and T2D are needed. The completion of these studies will better define how the MCU complex may impinge on β-cell associated pathologies observed in these different physiological states. 

## Figures and Tables

**Table 1:** Known MCU complex components and their function in the pancreatic β-cell.

MCU Complex Component	Function in the β-Cell
MCU protein	Essential for GSIS [35] May alleviate T2D symptoms [36]
MICU1	Promotes mitochondrial Ca2+ influx [35], May play a role in apoptosis regulation [37]
MICU2	Essential for sustained GSIS [38]
MICU3	N/A
EMRE	Positioning facilitates rapid GSIS following an excitatory stimulus [39]

**Table 2:** Known MCU complex mutations with associated phenotype.

Mutated Protein	Molecular Effect	Clinical Phenotype
MCU	Decreased apoptosis [35], decreased pyruvate dehydrogenase activity [77]	Increased levels of spontaneous abortion [79], cardioprotective effects [80]
MICU1	Decreased pyruvate dehydrogenase activity [82]	Decreased insulin secretion [82] extrapyramidal proximal myopathy [83]
MICU2	No known molecular effect	Severe neurological disorder and cognitive impairment [84]
MICU3	No known molecular effect	No known clinical phenotype
EMRE	No known molecular effect	probable spontaneous abortion [85]
